# Supplementary figures and images for: Antiviral Activity of Water–Alcoholic Extract of Cistus incanus L
Source: Int J Mol Sci. 2025 Jan 23;26(3):947. doi: 10.3390/ijms26030947 (PMC11817444; doi:10.3390/ijms26030947)

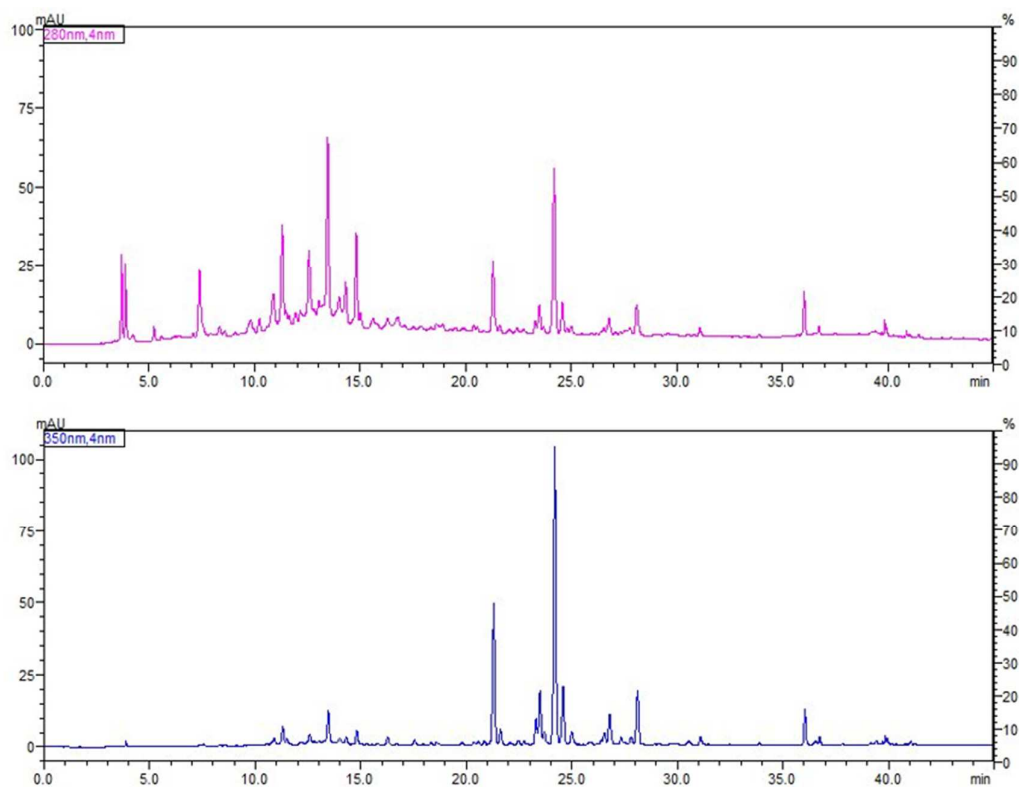

**Figure S1:** HPLC-PDA chromatograms at 280 nm (upper) and at 350 nm (down) of *C. incanus* L. extract

Supplement: Supplementary file 1 [file ijms-26-00947-s001.zip › ijms-3425490-supplementary.pdf]
